# The influence of assistive technology and home modifications on falls in community-dwelling older adults: a systematic review protocol

**DOI:** 10.1186/s13643-023-02354-7

**Published:** 2023-11-07

**Authors:** Kathryn M. Crosby, Catarina A. Rodriguez, Matthew A. Canas, Changki Kim, Shamim Noroozi, Mathew Vis-Dunbar, Vicki Komisar, Brodie M. Sakakibara, Jennifer M. Jakobi

**Affiliations:** 1 Aging in Place Research Cluster, University of British Columbia Okanagan, 233-1147 Research Road (Arts Building), Kelowna, BC V1V 1V7 Canada; 2 School of Health & Exercise Sciences, University of British Columbia Okanagan, Kelowna, BC Canada; 3 School of Engineering, University of British Columbia Okanagan, Kelowna, BC Canada; 4 Okanagan Library, University of British Columbia Okanagan, Kelowna, BC Canada; 5 Centre for Chronic Disease Prevention and Management, University of British Columbia Okanagan, Kelowna, BC Canada; 6 Department of Occupational Science and Occupational Therapy, University of British Columbia, Vancouver, BC Canada

**Keywords:** Systematic review protocol, Fall prevention, Mobility aids, Assistive technologies, Aging, Home modifications

## Abstract

**Background:**

Fall-related injuries can reduce older adults’ independence and result in economic burdens. The assistive technologies and home modifications explored in this review are suggested to reduce the risk of falls of community-dwelling older people. However, the location of the in-home assistive technology being used, and the in-home modification likely interact and influence fall reduction and injury prevention of community-dwelling older adults. This interactive effect is poorly understood. A better understanding of the impact of assistive technologies and modifications in the homes of older adults is needed to support the appropriate application of these devices.

**Objective:**

The objective of this systematic review is to detail the contribution of assistive technology and home modification on falls, fall frequency, fall severity, and fall location within the homes of community-dwelling older adults.

**Methods:**

We will source articles from 3 databases (MEDLINE, CINAHL, Web of Science Core Collection) and will assess them using a set of pre-defined inclusion and exclusion criteria. Reporting will be in accordance with PRISMA 2020. Two independent reviewers will screen each study at the title and abstract and full-text level. We are managing citations within the Covidence software. Data extraction and analysis will be reported in a systematic review.

**Discussion:**

The outcome variables of interest are fall frequency, fall location, injury, mortality, and hospitalization. These variables of interest all relate to falls, their severity, and their locations in the home. We are seeking a better understanding of how these outcomes vary with the use of different assistive technologies and home modifications as reported in the literature. This will help us understand where falls occur which may inform how different assistive technologies can be used by community-dwelling older adults to prevent falls and adverse outcomes in different areas of their homes. Our review will provide a basis for more intentional prescription of ambulatory assistive technologies and evidence-based recommendations of home modifications. It may also inform adaptations to existing technologies to foster safer mobility in the homes of community-dwelling older adults.

**Systematic review registration:**

This protocol has been submitted for registration in PROSPERO CRD42022370172 on October 24, 2022.

## Background

In Canada and many other areas of the world, older adults are the fastest-growing population [1]. The percentage of the world’s population aged 60 and over is predicted to nearly double between 2015 and 2050, from 12 to 22% [2]. Among older adults, falls are the leading cause of injury-related hospitalizations in Canada, 44% of falls occur inside the home and fall-related injuries reduce older adults’ independence and result in a large economic burden [3–5]. A systematic review on fall prevalence worldwide reported that 26.5% of older adults experience a fall, and the highest rates of prevalence are in Oceania (34.4%) and America (27.9%) [6]. To prevent the large number of in-home falls, various assistive technologies (e.g., grab bars, bathtub support, rails, toilet lift seat, shower stool, canes, walkers, and stair lift) are recommended.

Previous studies have explored the use of assistive technologies, including environmental modifications for fall reduction both independently and as part of multifactorial interventions [7–9]. However, the impacts of specific technologies or modifications in various areas of community-dwelling older adults’ homes for preventing falls and adverse outcomes (e.g., fracture, hospitalization, mortality) are poorly understood. Certain assistive devices may be more effective at preventing falls and adverse events than others, and their efficacy may vary based on which area of the home they are used in due to interactions with environmental hazards and human behaviors. Previous systematic reviews [10] have investigated the effects of overall home safety modifications on falls in community-dwelling older adults, but this review will be the first to investigate, with identification and delineation of the specific assistive technologies (e.g., the effects of only grab bar implementation) on falls [11]. This is not addressed in either the Clemson and colleagues (2023) or Gillespie and colleagues [11] reviews. We aim to identify the specific technology (e.g., grab bar implementation) rather than global adaptations (e.g., in-home modifications) and the most effective assistive technologies implemented in the home environment to work towards cost-effective and targeted fall prevention. Our systematic review differs from the recent Clemson and colleagues (2023) review in other ways. Clemson and colleagues (2023) included a broader scope of intervention, including special footwear, prescription glasses, and behavioral strategies to reduce in-home falls. The present study’s inclusion criteria focus solely on assistive technologies that support in-home mobility and functional independence. Therefore, the results of this study will be more specific to assistive technologies used to alter the in-home environment. A better understanding of the impact of assistive technologies use in the homes of older adults is needed to support the appropriate application of these devices. Informed use of assistive devices can reduce adverse fall-related events and support functional independence, helping community-dwelling older adults to age in place.

## Methods

### Objectives and research questions

The purpose of this systematic review is to examine the influence of home modifications, and the type and location of assistive technologies used on fall-related adverse outcomes in community-dwelling older adults within their homes. Our primary research question is what is the influence of various assistive technologies and housing adaptations on fall frequency? We are also interested in the secondary research questions of (1) How do different assistive technologies affect fall location in the home? (2) How do different assistive technologies influence rates of fall-related fractures, hospitalizations, and mortalities?

### Study eligibility

Studies will be included if they investigate the effects of assistive technologies on falls in the homes of functionally independent community-dwelling older adults. Falls are defined as “inadvertently coming to rest on the ground, floor, or other lower level, excluding intentional change in position to rest on furniture, wall, or other objects.” [4].

No restrictions are being placed on publication date, language, or study duration. Studies will not be included if they are literature reviews, book chapters, conference abstracts, protocols, or books. All study types not listed previously are eligible to be included (e.g., randomized controlled trials, cohort studies, single-group pre-post studies, unpublished data from clinical trial database). Gray literature sources (e.g., policy literature, newsletters, government documents, speeches, urban plans) will be excluded.

Eligible study populations are community-dwelling men and women aged 60 years or over. Studies will be included if the intervention implements or studies assistive technologies in the home, including environmental (e.g., handrails, grab bar, shower chair) or ambulatory (e.g., canes, walkers) mobility aids, to assess the effects on fall-related outcomes. Studies must explicitly report on the specific assistive technology or home modification implemented. Multifactorial interventions which specify the assistive technologies or home modifications implemented will be included if they meet all other criteria.

Studies that take place in hospitals, long-term care facilities, nursing homes, and assisted living facilities are ineligible for review. Also, research studies that explore wearable technology, e.g., hip protectors, anti-slip shore covers, will be excluded. If the assistive technologies in the study intervention are not directly related to supporting mobility within the home (e.g., fall sensors) they will be excluded. Studies involving populations with clinical conditions (e.g., stroke, cancer, arthritis, neurological diseases, cardiovascular disease, Alzheimer’s disease, and limb amputation) without healthy control groups will also be excluded. If a clinical population study meets all other eligibility criteria and reports data for the non-diseased control group, inclusive of older adults with mild cognitive impairment co-morbidities of aging, e.g., frailty they will be included in the full-text review.

The main fall frequency variables of interest include the following:

1. Number of falls (number of falls recorded for the study population)
2. Number of fallers (individuals who fell at least once)
3. Fall rate (number of falls in the population per unit time)

Additional variables are as follows:

1. Number of recurrent fallers (individuals who experienced multiple reported falls)
2. Fall location within the home
3. Number of fall-related injuries
4. Number of falls resulting in fracture(s) (fractures sustained from a fall in the study population)
5. Number of falls involving hospitalizations (individuals hospitalized after a fall during the study period)
6. Number of fall-related mortalities (fall-related deaths in the population)

### Identify relevant studies

The databases being searched include MEDLINE (OVID, 1946-Present), CINAHL (EBSCOhost, 1961-Present), and Web of Science Core Collection (Web of Science, 1900-Present). ClinicalTrials.gov will be searched to identify relevant unpublished data. In addition to a formal search strategy, the authors will also employ snowballing techniques, following relevant citations from included studies to limit the possibility of omitting relevant published works. Study authors will be contacted when articles cannot be found by reviewers. The authors will be contacted if additional information is required. The authors will attempt to translate for inclusion all non-English studies that make it through title and abstract screening. Any non-English studies that cannot be translated will be retained to comprehensively document research in this field. Literature search results will be uploaded to Covidence for data management and inclusion/exclusion decisions. The literature search protocol was developed in consultation with a health sciences research librarian.

The MEDLINE search consisted of the three strands below put together with the Boolean search command “AND.”

((assessment* or adaptation* or modification* or safety or intervention?) adj3 (environmental or indoor? or home? or hous* or "community-dwelling" or (independent* adj living) or "living in the community")).ab.ti OR ((assessment* or adaptation* or modification* or safety or intervention?).ti,kw.

(environmental or indoor? or home? or hous* or "community-dwelling" or (independent* adj living) or "living in the community").ti,kw. OR (Assistive technolog* or Assistive device* or Assistive aid* or Ambulatory device*).ab,ti,kw. OR independent living/

("recurrent falls" or "repeat* falls" or "one or more falls" or (fall* adj2 risk) or (fall* adj2 injur*) or (fall* adj4 prevent*) or (prevalence adj2 fall*)).ab,ti,kw. OR accidental falls/

### Study selection

#### Screening

We will be using Covidence for title and abstract screening as well as full-text eligibility. All inclusion and exclusion decisions for the title and abstract and full-text screening phases will be documented in Covidence. Before screening, inter-rater reliability (IRR) will be measured between each reviewer using a select number of studies. Cohen’s Kappa coefficient will be calculated as a measure of interrater reliability (IRR) between each reviewer, aiming for a minimum of moderate level of agreement (Kappa value greater than 0.60). Screening will be done independently by four reviewers (KC, CR, SN, MC) with each study being screened by two individuals at both the title and abstract and full-text review stages. Decisions will be made based on predefined inclusion/exclusion criteria. Conflicts during the title and abstract screening and full-text eligibility will be resolved through a discussion between the two reviewers and if agreement cannot be reached, and a third reviewer will offer a discrepancy assessment.

#### Data extraction

Reviewers will extract data from all included studies independently using a study-specific data extraction table. The data extraction table will be piloted by all reviewers using select studies to ensure agreement. Two reviewers will extract data from the full paper of each research study. Any disagreements during the data extraction phase will be resolved through a discussion, and if a consensus cannot be reached, another reviewer will be engaged. The data items to be extracted will include the type of home (e.g., apartment, multi-storey home), study characteristics (e.g., authors, type, language, country, date of publication, number of participants, inclusion and exclusion criteria, length of follow-up), participant characteristics (e.g., age, sex, ethnicity, socioeconomic status, number and types of medications), intervention characteristics (e.g., assistive technologies used, home modifications made, study duration of monitoring or intervention, how outcomes were measured and reported), comparison characteristics (e.g., types of assistive technologies or home modifications, effectiveness of assistive technologies, or home modifications in specific locations), outcomes (e.g., number of falls, number of fallers, fall rate, fall location within the home, number of recurrent fallers, number of fall-related injuries, number of falls resulting in fracture(s) or hospitalizations, number of fall-related mortalities), study funding, and conflicts of interest. Study authors will be contacted to obtain incomplete data (e.g., standard deviation, effect size).

#### Quality assessment

Two reviewers will carry out a risk of bias assessment for each included study using the Cochrane Risk of Bias (RoB 2) Tool for randomized studies and the Cochrane Risk of Bias in Non-randomized Studies of Interventions (ROBINS-I) tool for non-randomized studies [12, 13]. A low risk of bias rating on the ROBINS-I tool indicates a high-quality study. A critical risk of bias rating indicates the study is too problematic to provide useful evidence [12]. Risk of bias will be assessed at the intervention level. Any inconsistencies in scores will be resolved with input from a third reviewer. Evidence will be synthesized with consideration to the risk of bias ratings of interventions in individual studies. The GRADE (Grading of Recommendations, Assessment, Development, and Evaluations) framework will be used to assess the certainty of evidence [14].

#### Data synthesis

PRISMA 2020 guidelines will be used for data synthesis and analysis.

All the data obtained from the studies will be summarized within the data extraction table created in Microsoft Excel Version 16.63 (Redmond, USA). There will be both a narrative (descriptive) summary of studies as well as a quantitative analysis of study results. Grouping of studies according to various variables (e.g., fall risk, frequency, prevalence, hospitalization, mortality, etc.) will be collated through the creation of pivot tables to facilitate numerical quantification. Continuous metric outcome variables (e.g., fall repeat) will be analyzed using central tendency metrics (mean or median) and spread around the mean (standard deviation or interquartile range). Effect sizes will be calculated using group means and standard deviations when feasible. The results will be reported following the 2020 Preferred Reporting Items for Systematic Reviews and Meta-Analyses (PRISMA).

## Discussion

The aim of this systematic review is to review the literature reporting on assistive technology use in the home of community-dwelling older adults on falls, and fall frequency, location, and hospitalization. We are seeking a better understanding of how fall factors change with the use of different assistive technologies and hope to identify the types of assistive technology most effective in preventing falls and fall-related adverse outcomes. Ultimately, our results will provide information on the influence of assistive technology use on fall prevention among older adults.

We anticipate that not all devices and home modifications will be equally effective at preventing falls and adverse outcomes in different areas of the home. The findings will help us understand how different assistive technologies can best be used by community-dwelling older adults to foster safer mobility in different areas of their homes. We hope our results will better inform the prescription of assistive devices and recommendations of in-home device use and in-house modifications for older adults. Devices without an evidence base may require improvements or new alternatives to reduce falls in the homes of community-dwelling older adults.

## Data Availability

Not applicable.
